# Efficacy and tolerability of bevacizumab plus capecitabine as first-line therapy in patients with advanced hepatocellular carcinoma

**DOI:** 10.1038/sj.bjc.6605580

**Published:** 2010-02-16

**Authors:** C-H Hsu, T-S Yang, C Hsu, H C Toh, R J Epstein, L-T Hsiao, P-J Chen, Z-Z Lin, T-Y Chao, A-L Cheng

**Affiliations:** 1 Department of Oncology, National Taiwan University Hospital, 7 Chung-Shan South Road, Taipei 10002, Taiwan; 2 Division of Haematology and Oncology, Departmet of Internal Medicine, Chang Gung Medical Foundation, LinKuo-Branch, 5, Fusing St., Gueishan Township, Taoyuan County 333, Taiwan; 3 Department of Medical Oncology, National Cancer Center, 11 Hospital Drive, 169610, Singapore, Singapore; 4 Department of Medicine, University of Hong Kong, Queen Mary Hospital, 102 Pokfulam Road, Hong Kong, China; 5 Department of Hematology and Oncology, Taipei Veterans General Hospital, No. 201, Sec. 2, Shih-Pai Road, Taipei 112, Taiwan; 6 Department of Internal Medicine, National Taiwan University Hospital, 7 Chung-Shan South Road, Taipei 10002, Taiwan; 7 Division of Hematology and Oncology, Tri-Service General Hospital, No. 325, Sec. 2, Chenggong Road, Neihu District, Taipei City 114, Taiwan

**Keywords:** bevacizumab, capecitabine, hepatocellular carcinoma

## Abstract

**Background:**

Molecularly targeted agents with anti-angiogenic activity, including bevacizumab, have demonstrated clinical activity in patients with advanced/metastatic hepatocellular carcinoma (HCC). This multicentre phase II study involving patients from several Asian countries sought to evaluate the safety and efficacy of bevacizumab plus capecitabine in this population.

**Methods:**

Histologically proven/clinically diagnosed advanced HCC patients received bevacizumab 7.5 mg kg^–1^ on day 1 and capecitabine 800 mg m^–2^ twice daily on days 1–14 every 3 weeks as first-line therapy.

**Results:**

A total of 45 patients were enrolled; 44 (96%) had extrahepatic metastasis and/or major vessel invasion and 30 (67%) had hepatitis B. No grade 3/4 haematological toxicity occurred. Treatment-related grade 3/4 non-haematological toxicities included diarrhoea (*n*=2, 4%), nausea/vomiting (*n*=1, 2%), gastrointestinal bleeding (*n*=4, 9%) and hand–foot syndrome (*n*=4, 9%). The overall response rate (RECIST) was 9% and the disease control rate was 52%. Overall, median progression-free survival (PFS) and overall survival (OS) were 2.7 and 5.9 months, respectively. Median PFS and OS were 3.6 and 8.2 months, respectively, for Cancer of the Liver Italian Programme (CLIP) score ⩽3 patients, and 1.4 and 3.3 months, respectively, for CLIP score 4 patients.

**Conclusion:**

The bevacizumab–capecitabine combination shows good tolerability and modest anti-tumour activity in patients with advanced HCC.

Primary liver cancer, which consists predominantly of hepatocellular cancer (HCC), is the fifth most common cancer worldwide and the third most common cause of cancer mortality (El-Serag and Rudolph, 2007). More than 80% of cases occur in East Asia and sub-Saharan Africa, although the incidence is increasing in parts of Europe (Capocaccia *et al*, 2007) and the United States (El-Serag and Rudolph, 2007). Patients with locally advanced or metastatic HCC face a dismal outcome. Conventional chemotherapy is rarely successful and often poorly tolerated (Palmer *et al*, 2004; Zhu, 2006a). In the past, the median survival for these patients diagnosed in Asia was typically 2–4 months with best supportive care (Hsu *et al*, 2010).

Recently, sorafenib, a multikinase inhibitor with anti-angiogenic activity (Wilhelm *et al*, 2004, 2006), demonstrated a survival benefit *vs* best supportive care in patients with advanced HCC in two randomised placebo-controlled trials, one conducted in Europe, North America, South America and Australasia (the SHARP study, Llovet *et al*, 2008b) and the other in the Asia-Pacific region (Cheng *et al*, 2009). Both trials reported similar objective response rates (2–3%) and disease control rates (35–43%); overall survival (OS) was 10.7 months in the SHARP trial (Llovet *et al*, 2008b) and 6.5 months in the Asia-Pacific trial (Cheng *et al*, 2009). Although the survival benefit conferred by sorafenib in advanced HCC is an encouraging development, there is still a substantial unmet medical need for effective treatment options with favourable safety profiles in this difficult-to-treat population.

Hepatocellular carcinomas are known to be highly vascularised, with elevated vascular endothelial growth factor (VEGF) and microvessel density levels (Yamaguchi *et al*, 1998; Pang and Poon, 2006). The VEGF and VEGFR signalling pathways are the prime targets for developing anti-angiogenic therapy as cancer therapeutics. Bevacizumab is an anti-VEGF antibody that targets tumour angiogenesis and has proven benefit in many solid tumours (Hurwitz *et al*, 2004; Miller *et al*, 2005; Sandler *et al*, 2006); bevacizumab has also been investigated in advanced HCC in a phase II setting. As a single agent, bevacizumab had an objective tumour response rate of 13% in patients with unresectable non-metastatic HCC (Siegel *et al*, 2008). The combination of bevacizumab with standard chemotherapy – a strategy that has demonstrated clinical benefit in patients with colorectal, lung and breast cancer (Hurwitz *et al*, 2004; Miller *et al*, 2005; Sandler *et al*, 2006) – has also been tested in patients with HCC. In phase II studies, the combination of bevacizumab with gemcitabine–oxaliplatin (GEMOX) (Zhu *et al*, 2006b) or capecitabine–oxaliplatin (XELOX) (Sun *et al*, 2007) resulted in response rates of up to 20%, although these regimens were associated with significant treatment-related toxicity. To have a wider application, bevacizumab combinations with a better therapeutic index need to be developed.

Capecitabine, an oral fluoropyrimidine with a favourable safety profile, has been widely used in various types of solid cancers (Roche Products Limited, 2009). Theoretically, capecitabine may have an advantage when treating patients with an abnormal liver function. Indeed, Twelves *et al* (1999) demonstrated that mild to moderate hepatic dysfunction has no clinically significant influence on the pharmacokinetic parameters of capecitabine and its metabolites. In a retrospective analysis, capecitabine monotherapy resulted in a response rate of 11% in patients with advanced HCC (Patt *et al*, 2004).

The combination of bevacizumab with capecitabine, two drugs that have previously demonstrated single-agent activity in patients with advanced HCC, has not yet been evaluated in patients with advanced HCC. The present phase II study was conducted at multiple Asian centres to evaluate the tolerability and efficacy of bevacizumab plus capecitabine in patients with advanced or metastatic HCC.

## Patients and methods

### Patients

Patients aged ⩾18 years were included if they had histologically confirmed or clinically diagnosed HCC (typical imaging findings plus *α*-fetoprotein ⩾400 ng ml^–1^); stage IV disease (American Joint Committee on Cancer, 1998) that was inoperable and not amendable by other locoregional therapies; Child–Pugh class A; Karnofsky performance status ⩾70% ⩾1 measurable lesion; neutrophil count ⩾2000 per *μ*l; platelet count ⩾150 000 per *μ*l; alanine aminotransferase level ⩽5 × upper normal limit (UNL); or bilirubin ⩽1.2 × UNL. Exclusion criteria included earlier radiotherapy/systemic therapy for advanced HCC; central nervous system metastases; previous HCC rupture; and clinically significant cardiovascular disease. Patients with a history of gastrointestinal bleeding within 1 year or known oesophageal/gastric varices were required to undergo gastroduodenoscopy to exclude active bleeding and a high risk of bleeding.

The study conformed to the principles of the Declaration of Helsinki and Good Clinical Practice Guidelines, with approval obtained from each centre's independent ethics committee. Patients provided written informed consent.

### Treatment

Patients received intravenous bevacizumab 7.5 mg kg^–1^ on day 1 and oral capecitabine 800 mg m^–2^ twice daily on days 1–14 every 3 weeks. The dose of capecitabine was selected on the basis of several observations indicating that capecitabine given in lower doses demonstrated an improved therapeutic index (Sakamoto *et al*, 2004; Hennessy *et al*, 2005). For example, in a Japanese cohort of advanced colorectal cancer patients, capecitabine at a dose of 828 mg m^–2^ twice daily resulted in an improved toxicity profile without compromising its anti-tumour activities (Sakamoto *et al*, 2004). Six treatment cycles were planned, but patients maintaining a response or stable disease after six cycles could continue treatment at the investigator’s discretion.

No dose reduction of bevacizumab was allowed, except for patients with >10% change in body weight during the treatment period. Bevacizumab was discontinued in patients with gastrointestinal perforation, arterial thrombotic toxicity, grade 3/4 haemorrhagic toxicity, symptomatic grade 4 thromboembolic toxicity, hypertensive crisis or nephrotic syndrome. Bevacizumab was withheld for grade 3 hypertension and repeated 24 h proteinuria >2 g until these symptoms improved. Capecitabine doses were reduced by 20% for patients who experienced a first occurrence of a grade 3 haematological toxicity, a second occurrence of grade 2 non-haematological toxicity or any grade 3 non-haematological toxicity. Capecitabine doses were reduced by 40% for a first occurrence of grade 4 haematological toxicity, a second occurrence of grade 3 haematological toxicity, any grade 4 non-haematological toxicity, a second occurrence of grade 3 non-haematological toxicity or a third occurrence of grade 2 non-haematological toxicity. Capecitabine was discontinued after the occurrence of a second grade 4 haematological toxicity, a third grade 3 haematological toxicity, a fourth grade 2 non-haematological toxicity, a third grade 3 non-haematological toxicity or a second grade 4 non-haematological toxicity.

### Assessments

Tumour assessment according to the Response Evaluation Criteria in Solid Tumors (RECIST) was performed every 6 weeks using computed tomography/magnetic resonance imaging. Adverse events (AEs) were graded using National Cancer Institute Common Terminology Criteria for Adverse Events, version 3 (National Cancer Institute, 2008).

### Statistical analyses

The null hypothesis (H0) was that the overall response rate (ORR) was ⩽10%, that is, low activity. The alternative hypothesis (H1) was that ORR was ⩾25%, that is, encouraging activity. To distinguish between an ORR of 10 and 25%, assuming *α*- and *β*-error rates of 0.05 and 0.20, respectively, a sample size of 43 patients was required using Ensign's three-stage design (Ensign *et al*, 1994). Stage 1 enrolled nine patients and would stop if no responses were confirmed. Stage 2 would enrol 25 patients (including the nine patients from stage 1) and would stop if ⩽three responses were confirmed. If sufficient responses were observed in stages 1 and 2, the trial proceeded to stage 3, recruiting 45 patients in total.

The primary objective of this study was to evaluate the ORR for capecitabine plus bevacizumab in patients with advanced/metastatic HCC. ORR was assessed in the per-protocol population, which excluded patients who did not have adequate baseline or ⩾1 follow-up tumour assessment. Secondary objectives included safety, disease control rate, progression-free survival (PFS) and OS. Secondary analyses were performed on the intent-to-treat population. The PFS and OS were analysed using Kaplan–Meier plots and presented as median event times with 95% confidence intervals (CIs).

## Results

### Patients

Between May 2005 and August 2006, 45 Asian patients were enrolled at eight centres in Taiwan, Singapore and Hong Kong. Patients’ baseline characteristics are shown in Table 1. All patients except one had extrahepatic metastases (*n*=31) and/or major hepatic vessel invasion (*n*=25): thus 44 patients (98%) had Barcelona Clinic Liver Cancer (BCLC) stage C cancer and one had BCLC stage B disease. The most common disease sites were the liver (91%), lung (47%) and lymph nodes (31%).

Patients received a median of three cycles (range 1–31 cycles) of bevacizumab plus capecitabine: 38 patients (87%) completed two cycles, 21 (47%) completed four cycles and 15 (33%) completed six cycles. Among these 15 patients, seven continued study treatment for between 10 and 31 cycles. The reasons for discontinuation before six cycles were progressive disease in 25 patients, safety concern in four patients (fulminant hepatitis, gastrointestinal bleeding, hypoalbuminaemia and hyponatraemia, respectively), and withdrawal of consent in one patient. The capecitabine dose was reduced in five patients (elevated bilirubin, *n*=1; hand–foot syndrome, *n*=2; grade 3 diarrhoea, *n*=1; vomiting, *n*=1). The bevacizumab dose was delayed in three patients as a result of capecitabine toxicity and adjusted in one patient because of weight loss.

### Efficacy

One patient with no follow-up tumour assessment was excluded from the per-protocol population; 44 patients were therefore evaluable for response. Four patients had a partial response and 19 had stable disease for an ORR of 9% and a disease control rate of 52% (95% CI: 36.7–67.5% Table 2).

Serum alpha-fetoprotein (AFP) levels were serially monitored. A total of 37 patients who had elevated baseline AFP were eligible for AFP response. AFP response was 24% if defined by 20% decline from baseline (Chan *et al*, 2009) or 13% if defined by 50% decline (Chen *et al*, 2005; Vora *et al*, 2009).

**Figure 1:**
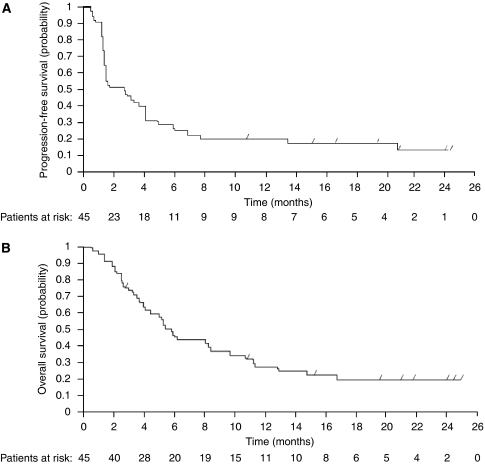
Duration of progression-free survival (**A**) and overall survival (**B**) in patients treated with capecitabine and bevacizumab (*n*=45).

After a median follow-up of 19.6 months, the median PFS was 2.7 months (95% CI: 1.5–4.1 months) and median OS was 5.9 months (95% CI: 4.1–9.7 months) in the intent-to-treat population (Figure 1). The 1-year PFS rate was 20% (95% CI: 8–32%) and 1-year OS was 27% (95% CI: 14–40%). Correlations between several baseline characteristics and PFS/OS were explored. Only Cancer of the Liver Italian Programme (CLIP) score was significantly correlated with outcome: median PFS was higher in patients with CLIP scores ⩽3 compared with those with a score of 4 (3.6 months (95% CI: 1.5–6.0 months) *vs* 1.4 months (95% CI: 1.2–3.3 months)), whereas median OS was 8.2 months (95% CI: 5.0–11.3 months) *vs* 3.3 months (95% CI: 2.5–5.2 months), respectively.

### Tolerability

All 45 patients were evaluable for tolerability. The combination of bevacizumab plus capecitabine was generally well tolerated. Grade 3/4 AEs and laboratory abnormalities are shown in Table 3. In total, 43 patients (96%) experienced ⩾1 AEs; 25 patients (56%) had AEs that were possibly related to treatment; and 26 patients (58%) had AEs that were probably related to treatment. Treatment-related grade 3/4 toxicities were diarrhoea (*n*=2, 4%), nausea/vomiting (*n*=1, 4%), gastrointestinal bleeding (*n*=4, 9%, including three patients with oesophageal variceal bleeding), hand–foot syndrome (*n*=4, 9%), lower respiratory tract infection (*n*=1, 2%) and proteinuria (*n*=1, 2%).

A total of 15 patients had serious AEs; most were gastrointestinal in nature (*n*=9). Three patients withdrew early from the study as a result of AEs. No deaths were attributed to the study treatment.

## Discussion

Targeted therapy with anti-angiogenic activity has become the mainstay of systemic therapy for advanced HCC (National Comprehensive Cancer Network, 2007). Previous studies have shown that bevacizumab is also effective in this difficult-to-treat indication (Zhu *et al*, 2006b; Sun *et al*, 2007; Siegel *et al*, 2008). Further evidence of this activity has been demonstrated in this study. However, the study did not meet the hypothesis that ORR would be ⩾25%, although ORR, combined with PFS and OS, suggests activity for this combination. Retrospectively, it has been recognised that ORR may not be an informative end point in studies of targeted agents for advanced HCC (Llovet *et al*, 2008a).

At first glance, the efficacy results reported here are lower than those previously reported in bevacizumab–chemotherapy combination trials (Zhu *et al*, 2006b; Sun *et al*, 2007). Nevertheless, comparisons between studies of advanced HCC are complicated by differences in recruitment criteria and study designs, as well as by differences in baseline patient characteristics, in particular the proportion of Asian patients in the studies. This is illustrated by two recent sorafenib studies: the SHARP study, which enrolled patients exclusively from Europe and North America (Llovet *et al*, 2008b), and the Asia-Pacific study, which enrolled patients exclusively from China, Korea and Taiwan (Cheng *et al*, 2009). Both studies used the same eligibility criteria and were conducted in parallel; although clinical improvement was comparable in the two studies, overall outcomes were much poorer in patients in the Asia-Pacific study. Specifically, median OS was 4.2 and 6.5 months for placebo- and sorafenib-treated patients in the Asia-Pacific study, respectively, compared with 7.9 and 10.7 months in the SHARP study. Notably, previously reported clinical studies of bevacizumab with or without chemotherapy in advanced HCC patients were all reported from North America (Zhu *et al*, 2006b; Sun *et al*, 2007; Siegel *et al*, 2008). Taking the advanced HCC patients enrolled in the Asia-Pacific study of sorafenib as a reference group (Cheng *et al*, 2009), the efficacy outcomes of the current combination are comparable with those of sorafenib.

The efficacy of the bevacizumab plus capecitabine combination is also likely to have been influenced by the fact that this study included a large proportion of poor-prognosis patients. Almost a quarter of patients had a CLIP score of 4, which is associated with poor prognosis (Farinati *et al*, 2000; Grieco *et al*, 2005; Collette *et al*, 2008). In a study of survival in patients undergoing palliative treatment, those with CLIP scores of 2–3 had a median OS of 4.57 months, whereas those with CLIP scores of 4–6 had a median OS of 1.93 months (Collette *et al*, 2008). This pattern was also apparent in the present analysis of survival: patients with a CLIP score ⩽3 had a median PFS of 3.6 months and an OS of 8.2 months compared with 1.4 months and 3.3 months, respectively, for patients with a CLIP score of 4. These data suggest that CLIP score is an important stratification factor for clinical trials in advanced HCC.

The combination of bevacizumab plus capecitabine was well tolerated in this group of patients with advanced HCC. Grade 3/4 haematological toxicities were not observed and the rate of other grade 3/4 toxicities was low. Gastrointestinal bleeding occurred in four patients, three of whom had oesophageal variceal bleeding. Mandatory gastroduodenoscopy was later incorporated into the screening phase for this study. Other bevacizumab HCC studies also adopted measures to exclude patients at high risk of bleeding (Siegel *et al*, 2008; Thomas *et al*, 2009). For example, in the study by Siegel *et al* (2008), the protocol was amended after a variceal bleed that led to the death of one patient in the safety evaluation phase; the amendment required subsequent patients who had varices before or evidence of varices on computed tomography/magnetic resonance imaging to undergo endoscopy within 4 weeks of study entry (Siegel *et al*, 2008).

The favourable toxicity profile of our regimen, compared with other combinations used in HCC, is primarily because of the low toxicity of reduced-dose capecitabine. For comparison, the combination of bevacizumab plus GEMOX was associated with grade 3/4 neutropaenia and thrombocytopaenia in 42 and 9% of patients, respectively (Zhu *et al*, 2006b). Bevacizumab plus XELOX was associated with grade 3/4 neutropaenia and thrombocytopaenia in 6 and 12% of patients, respectively, and with grade 3/4 peripheral neuropathy in 12% of patients (Sun *et al*, 2007). More stringent inclusion criteria adopted in our study, including higher levels of baseline neutrophil and platelet, may also contribute to the negligible haematological toxicity in our cohort.

The significance of adding capecitabine to bevacizumab in advanced HCC patients could not be evaluated in the current single-arm study. The single-agent response rate of bevacizumab, as reported by Siegel *et al* (2008), was 13%, which was even higher than that of our combination. However, the high response rate of bevacizumab shown in that report could be associated with the following facts: the study excluded patients with extrahepatic metastases; it enrolled patients with different aetiological factors; and used higher doses of bevacizumab in two-thirds of their patients. Further studies are warranted to identify the optimal dose of bevacizumab, as well as more effective combinations of bevacizumab in HCC (Thomas *et al*, 2009).

In summary, the combination of bevacizumab plus capecitabine shows good tolerability and modest anti-tumour activity in patients with advanced or metastatic HCC. Randomised trials are required to determine whether the combination of chemotherapy and bevacizumab is superior to treatment with bevacizumab alone.

## Figures and Tables

**Table 1:** Patient characteristics at baseline (*n*=45)

**Characteristic**	**Value**
*Sex,* n (%)	
Male	40 (89)
Female	5 (11)
Median age, years (range)	54 (23–75)
	
*Karnofsky performance status,* n (%)	
90–100	36 (80)
80	9 (20)
	
*Baseline α-fetoprotein,* n (%)	
⩾400 ng ml^–1^	32 (71)
<400 ng ml^–1^	13 (29)
	
*AJCC stage,*^a^ n (%)	
IVa	16 (36)
IVb	29 (64)
	
*BCLC stage,* n (%)	
B	1 (2)
C	44 (98)
	
*CLIP score,* n (%)	
⩽2	18 (40)
3	16 (36)
4	11 (24)
	
*Okuda score,* n (%)	
I	21 (47)
II	24 (53)
	
*Site of extrahepatic metastases,* n (%)	
Lung	21 (47)
Lymph nodes	14 (31)
	
*Prior curative surgery or locoregional therapy,* n (%)	
Yes	11 (24)
No	34 (76)
	
*Virology of underlying liver disease,* n (%)	
HBV	30 (67)
HCV	8 (18)
Non-B+non-C	10 (22)

Abbreviations: BCLC=Barcelona Clinic Liver Cancer; CLIP=Cancer of the Liver Italian Programme; HBV=hepatitis B virus; HCV=hepatitis C virus.

a American Joint Committee on Cancer (1998) staging system.

**Table 2:** Efficacy of bevacizumab plus capecitabine in patients with hepatocellular carcinoma (*n*=44)

**Outcome**	**Value**
Overall response rate (95% CI), %	9.1 (2.5–21.7)
Complete response, *n* (%)	0 (0)
Partial response, *n* (%)	4 (9.1)
Stable disease, *n* (%)	19 (43)
Progressive disease, *n* (%)	21 (48)
Disease control rate (95% CI), %	52.3 (36.7–67.5)

Abbreviation: CI=confidence interval.

**Table 3:** Adverse events in patients with hepatocellular carcinoma treated with capecitabine plus bevacizumab (*n*=45)

	**No. of patients (%)**		
**Event**	**All grades**	**Grade 3**	**Grade 4**
*Adverse events*			
Hand–foot syndrome	15 (33)	4 (9)	0 (0)
Nausea	7 (16)	1 (2)	0 (0)
Vomiting	6 (13)	1 (2)	0 (0)
Diarrhoea	12 (27)	2 (4)	0 (0)
Gastrointestinal bleeding	4 (9)	2 (4)	2 (4)
Proteinuria	2 (4)	1 (2)	0 (0)
Lower respiratory tract infection	1 (2)	1 (2)	0 (0)
Fulminant hepatitis	1 (2)	0 (0)	1 (2)
Mucositis	5 (11)	0 (0)	0 (0)
Skin pigmentation	3 (7)	0 (0)	0 (0)
			
*Laboratory values*			
Anaemia	12 (27)	2 (4)	2 (4)
Neutropenia	1 (2)	0 (0)	0 (0)
Thrombocytopenia	12 (27)	0 (0)	0 (0)
Increased hepatic transaminases			
ALT	12 (27)	0 (0)	1 (2)
AST	20 (44)	6 (13)	1 (2)
Hyperbilirubinaemia	30 (67)	5 (11)	0 (0)

Abbreviations: ALT=alanine aminotransferase; AST=aspartate aminotransferase.
